# No associations of a set of SNPs in the Vascular Endothelial Growth Factor (VEGF) and Matrix Metalloproteinase (MMP) genes with survival of colorectal cancer patients

**DOI:** 10.1002/cam4.796

**Published:** 2016-06-23

**Authors:** Lydia A. Dan, Salem Werdyani, Jingxiong Xu, Konstantin Shestopaloff, Angela Hyde, Elizabeth Dicks, Ban Younghusband, Jane Green, Patrick Parfrey, Wei Xu, Sevtap Savas

**Affiliations:** ^1^ Discipline of GeneticsFaculty of MedicineMemorial UniversitySt. John'sNewfoundlandCanada; ^2^ Department of BiostatisticsPrincess Margaret HospitalUniversity of TorontoTorontoOntarioCanada; ^3^ Dalla Lana School of Public HealthUniversity of TorontoTorontoOntarioCanada; ^4^ Clinical Epidemiology UnitFaculty of MedicineMemorial UniversitySt. John'sNewfoundlandCanada; ^5^ Discipline of OncologyFaculty of MedicineMemorial UniversitySt. John'sNewfoundlandCanada

**Keywords:** Angiogenesis, colorectal cancer, lymph‐angiogenesis, matrix metalloproteinases, metastasis, overall survival, prognosis, SNPs, vascular endothelial growth factors, VEGFs

## Abstract

In this study, we aimed to investigate the associations of genetic variations within select genes functioning in angiogenesis, lymph‐angiogenesis, and metastasis pathways and the risk of outcome in colorectal cancer patients. We followed a two‐stage analysis: First, 381 polymorphisms from 30 genes (eight Vascular Endothelial Growth Factor (VEGF) and 22 Matrix Metalloproteinase [MMP] genes) were investigated in the discovery cohort (*n* = 505). Then, 16 polymorphisms with the lowest *P*‐value in this analysis were investigated in a separate replication cohort (*n* = 247). Genotypes were obtained using the Illumina^®^ HumanOmni‐1‐Quad (discovery cohort) and Sequenom MassArray^®^ (replication cohort) platforms. The primary outcome measure was overall survival (OS). Kaplan–Meier, univariate and multivariable Cox regression methods were used to test the associations between genotypes and OS. Four SNPs (rs12365082, rs11225389, rs11225388, and rs2846707) had the univariate analysis *P* < 0.05 in both the discovery and replication cohorts. These SNPs are in linkage disequilibrium with each other to varying extent and are located in the *MMP8* and *MMP27* genes. In the multivariable analysis adjusting for age, stage, and microsatellite instability status, three of these SNPs (rs12365082, rs11225389, rs11225388) were independent predictors of OS (*P* < 0.05) in the discovery cohort. However, the same analysis in the replication cohort did not yield statistically significant results. Overall, while the genetic variations in the VEGF and MMP genes are attractive candidates as prognostic markers, our study showed no evidence of associations of a large set of SNPs in these genes and overall survival of colorectal cancer patients in our study.

## Introduction

Survival outcomes in colorectal cancer patients may be modified by a variety of factors, including genetic factors. Genetic variations of the genes that are biologically related to tumor progression and cancer‐related death, such as those acting in angiogenesis, lymph‐angiogenesis and metastasis pathways, are candidate prognostic biomarkers.

Angiogenesis (i.e., growth of new blood vessels) is a vital process with roles in development, reproduction (e.g., menstrual cycle), and tissue repair (e.g., wound healing). Abnormalities in angiogenesis during carcinogenesis can lead to neovascularization that can facilitate local tumor growth, invasion, and disease progression. Similarly, lymph‐angiogenesis (i.e., growth of new lymph vessels) is essential for metabolism as well as proper immune system function; however, in cancer, it helps with dissemination and increased metastatic capacity of tumor cells. The vascular endothelial growth factors (VEGFs) and their receptors (VEGFRs) are key players in these two pathways 1, 2, 3. Among the VEGF genes, *VEGFA* seems to be a hot research topic, expression and polymorphisms of which are frequently studied by cancer researchers 4, 5, 6, 7. A number of polymorphisms exist in or around the *VEGFA* gene, among which five polymorphisms are worth mentioning: T‐1498C T/C (also called ‐460T/C; rs833061), ‐1154G/A (rs1570360), and ‐2578C/A (rs699947) in the promoter region, ‐634G/C in the 5′‐UTR (also called +405 G/C; rs2010963), and +936C/T in the 3′‐UTR (rs3025039). A meta‐analysis has found that the minor allele of one of these polymorphisms, +405G>C (rs2010963), was associated with better survival in different cancers 8. These and other findings 9, 10, 11 show the importance of VEGF genes in cancer mortality and biomarker research.

Following angiogenesis/lymph‐angiogenesis, metastasis (i.e., movement of cancer cells via blood or lymphatic circulation and formation of secondary tumors at distant organs) is likely to occur. A number of genes and gene families have roles in this process. Among these, genes encoding the matrix metalloproteinases (MMPs) are well‐studied. MMPs are a family of endopeptidases with multi‐faceted roles and best known for their ability to degrade the components of the extracellular matrix such as collagen, gelatin, and fibronectin. Because of this function, MMPs are linked to many phenotypes, such as neurological conditions 12 and inflammatory bowel disease 13. In cancer, MMPs have two important roles: they help with the metastasis of cancer cells (through manipulating the extracellular matrix) and some MMPs also have proangiogenic and/or anti‐angiogenic roles 14. These functions of MMPs make them critical in metastatic disease 14, 15. Although they are not studied as intensely as the VEGFs, a limited number of studies have evaluated and suggested a role for the MMP genes as prognostic biomarkers 16, 17, 18, 19, 20.

Together with the fact that tumor invasion and metastasis are responsible for the majority of the cancer‐related deaths, previous findings suggest the importance of the genes acting in angiogenesis, lymph‐angiogenesis, and metastasis processes in patient survival. The objective of this study was to test association of survival outcomes in colorectal cancer patients and genetic polymorphisms from five VEGF ligand genes (*VEGFA, VEGFB, VEGFC, VEGFD, PGF*), three VEGF receptor genes (*FLT1, KDR, FLT4*), and 22 human matrix metalloproteinase (MMP) genes that function in angiogenesis, lymph‐angiogenesis, or metastasis pathways. Three hundred and eighty‐one SNPs were first examined in relation to overall survival in the discovery cohort of patients (*n* = 505). Sixteen SNPs with the lowest *P*‐values were then investigated in an additional cohort of colorectal cancer patients (replication cohort; *n* = 247). As an exploratory analysis, disease‐free survival analysis was also performed in the discovery cohort.

## Materials and Methods

### Patient cohorts

#### Discovery cohort

The discovery cohort was described previously 21. In short, it consisted of 505 Caucasian patients recruited to the Newfoundland Colorectal Cancer Registry (NFCCR). NFCCR collected 736 colorectal cancer patients diagnosed with this disease between 1999 and 2003 22, 23. Demographic, clinical and treatment‐related features as well as the outcome data were previously collected by this registry. The date of last follow up in this cohort was 2010 24. Among the 736 patients, the genomic DNAs (extracted from peripheral blood samples) were available for 539 patients; these patients were included in our large‐scale SNP genotyping experiments 21 (*please see below* ‐ SNP genotype data and selection of genes and polymorphisms).

#### Validation cohort

Patients in this cohort were diagnosed with colorectal cancer between 1998 and 1999 in Newfoundland 24. There were 280 patients with clinical data collected during this period. However, DNA samples were available only for 247 patients; these patients constituted the replication cohort. In this cohort, the DNA samples were extracted from either peripheral blood samples (*n* = 40) or the nontumor colon or rectum tissues obtained during the surgery (*n* = 207). Table 1 summarizes the baseline variables for this cohort.

**Table 1:** Baseline variables of the discovery and replication cohorts

Characteristic	Discovery cohort*n* (%)	Replication cohort*n* (%)
Sex		
Female	198 (39.2)	116 (47.0)
Male	307 (60.8)	131 (53.0)
Histology		
Nonmucinous	448 (88.7)	209 (84.6)
Mucinous	57 (11.3)	38 (15.4)
Location		
Colon	334 (66.1)	198 (80.2)
Rectum	171 (33.9)	49 (19.8)
Stage		
I	93 (18.4)	46 (18.6)
II	196 (38.8)	86 (34.8)
III	166 (32.9)	68 (27.5)
IV	50 (9.9)	40 (16.2)
Unknown	0 (0)	7 (2.8)
Grade		
Well/moderately differentiated	464 (91.9)	207 (83.8)
Poorly differentiated	37 (7.3)	37 (15.0)
Unknown	4 (0.8)	3 (1.2)
Vascular invasion		
Absent	308 (61.0)	n/a
Present	159 (31.5)	n/a
Unknown	38 (7.5)	n/a
Lymphatic invasion		
Absent	298 (59.0)	63 (25.5)
Present	167 (33.1)	99 (40.1)
Unknown	40 (7.9)	85 (34.4)
Familial risk		
Low risk	250 (49.5)	n/a
Moderate/high risk	255 (50.5)	n/a
MSI status		
MSI‐L/MSS	431 (85.3)	224 (90.7)
MSI‐H	53 (10.5)	23 (9.3)
Unknown	21 (4.2)	0 (0)
*Braf* Val600Glu mutation		
Absent	411 (81.4)	n/a
Present	47 (9.3)	n/a
Unknown	47 (9.3)	n/a
Adjuvant 5‐FU‐based chemotherapy status		
Not given	230 (45.5)	160 (64.8)
Given	261 (51.7)	69 (27.9)
Unknown	14 (2.8)	18 (7.3)
Adjuvant radiotherapy status		
Not given	364 (72.1)	n/a
Given	124 (24.6)	n/a
Unknown	17 (3.4)	n/a

The median age for the discovery and validation cohorts were 61.43 years (range: 20.7–75) and 68.76 years (range: 25.3–91.6), respectively. 5‐FU, 5‐Fluorouracil; MSI‐H, microsatellite instability‐high; MSI‐L, microsatellite instability‐low; MSS, microsatellite stable; n, number; n/a, not available.

### Ethics statement

In the discovery cohort, patients or close relatives (if the patient had deceased) gave written informed consent prior to participation. The majority of the patients in the validation cohort were not consented to, however, the Human Research Ethics Authority (HREA) of Newfoundland has waived the need for consent for these patients. This particular study was approved by the HREA prior to the start of the study (#12.206, #10.133).

### SNP genotype data and selection of genes and polymorphism

#### Discovery cohort

Patient genomic DNAs were genotyped using the Illumina^®^ human Omni1‐Quad genome‐wide SNP genotyping platform (by the service provider Centrillion Bioscience, CA, USA) as part of a previous genome‐wide project. A series of quality control and inclusion–exclusion criteria were implemented on the genotype data; these were described in an earlier publication on the discovery cohort 21. In brief, patients who (1) had dis‐concordant sex information (based on the genetic data vs. the self‐reported sex); (2) had extreme mean heterozygosity rate; (3) had first, second, or third degree relatives in the cohort; and (4) had non‐Caucasian ancestry were excluded (total 24 patients). In the end, 505 out of 539 patients who satisfied the quality control and inclusion measures constituted the study cohort 21. Baseline clinical and pathological characteristics of the cohort is shown in Table 1.

For this project, 31 genes from angiogenesis, lymph‐angiogenesis, and metastasis pathways were selected. The hg19 genome coordinates of each gene were retrieved from the UCSC genome browser 25. This information was then used by the PLINK software 26 to retrieve the SNPs located within these gene regions and their associated information using the patient genotype files. During this step, the following quality control and inclusion measures were implemented: SNPs that deviated from Hardy–Weinberg Equilibrium (HWE; *P *≤* *0.0001), SNPs with >5% missing genotype data, and SNPs with minor allele frequencies (MAFs) <5% were excluded. As a result, a total of 381 common polymorphisms (380 substitutions and one indel polymorphism) located in 30 genes were identified. For simplicity, we refer to all of these polymorphisms as SNPs in this manuscript. Table S1 shows the list of selected genes and the number of SNPs/gene examined in this study; except *MMP23B* there was at least one SNP/gene examined. We note that, previously two of these polymorphisms, *VEGFA*_rs2010963 and *VEGFA*_rs3025039, were analyzed in relation to overall and disease‐free survivals in a NFCCR patient cohort highly similar to the discovery cohort patients 24.

#### Validation cohort

Sixteen SNPs that had the smallest *P*‐values in the univariate analysis of the discovery cohort were genotyped in the validation cohort patients using the Sequenom MassArray^®^ technique at a service provider (Clinical Genomics Centre, Mount Sinai Hospital, Toronto, Canada). Fifteen DNA samples were genotyped twice (6%) and in all cases the genotypes obtained were identical. All SNPs in this cohort had MAFs ≥5% and their genotype frequencies were in HWE equilibrium (calculated by R 27).

### Statistical analyses

#### Outcome measure

In this study, the endpoint of interest was death from any cause. The outcome measure, overall survival (OS), was defined as the time (in years) between the date of initial diagnosis of colorectal cancer and the date of death, or the last date of patient contact. We also performed an exploratory analysis for the disease‐free survival (DFS) in the discovery cohort, which was defined as the time from diagnosis of colorectal cancer till the time of recurrence, metastasis, or death (whichever occurred earlier).

In the discovery cohort, the number of events for OS and DFS were 170 (33.7%) and 200 (39.6%), respectively; event status was missing in the OS and DFS data (one patient each), and the remaining patients were event‐free at the time of last contact. The number of deaths in the validation cohort was 153 (62%). The median follow‐up time for OS in the discovery and validation cohorts were 6.36 years (range: 0.38–10.88) and 5.21 years (range: 0–12.48), respectively.

#### Prescreening of SNPs and selection of genetic models

The chances of finding an association are higher when a SNP is examined under the right genetic model 28. That is why we first prescreened the SNPs prior to statistical analyses and estimated appropriate genetic models for individual SNPs by constructing Kaplan–Meier survival curves under the codominant genetic model (where the patients were categorized into three groups based on their genotypes; AA = major allele homozygous, Aa = heterozygous, and aa = minor allele homozygous). Inspection of the Kaplan–Meier curves was then done (by L.A. D. and S.S) to estimate the best genetic model (dominant, recessive, codominant, or additive models) that may best fit a polymorphism 29. Specifically, for each SNP, the genetic model(s) that best fits the curve pattern and maximizes the chances of curve separation was estimated. When multiple genetic models were estimated for a SNP, the best genetic model was determined by univariate Cox regression analysis; whichever genetic model generated the lowest *P*‐value was deemed to be the best genetic model for that SNP. When there were not sufficient number of patients with the minor allele homozygous genotype (*n* ≤ 10), dominant genetic model was considered. A SNP was excluded from further analysis when the curves did not separate clear enough to estimate a genetic model or when they crossed each other at multiple times. This analysis excluded 91 and 72 SNPs from OS and DFS analyses, respectively. Of note, for each of the excluded SNPs, the log‐rank *P*‐values were >0.120 indicating that this prescreening step did not exclude SNPs with potential associations. For the interested readers, the Kaplan–Meier curves for 381 SNPs in OS and DFS analyses are shown in Tables S2–S7.

#### Survival analyses

Univariate Cox regression analysis was performed to test the association of clinical outcomes with (1) the SNPs under their estimated genetic models, and (2) the baseline clinicopathological, molecular, and treatment‐related features in the discovery cohort.

In the latter analysis, the variables that had a univariate analysis *P* < 0.05 were selected as baseline variables to construct multivariable models for OS and DFS separately; those variables that remained significant (*P* < 0.05) in these models were used to adjust for the genotypes during the multivariable analyses. Spearman's correlation test was performed prior to this analysis to determine whether the baseline variables investigated in the discovery cohort were correlated with each other; two variables were deemed to be highly correlated if the correlation score (r_s_) was ≥0.8. As a result of this analysis, lymphatic and vascular invasion (*r*_s_ = 0.963), and adjuvant chemotherapy and adjuvant 5‐FU‐chemotherapy status (*r*_s_  = 0.992) were found to be highly correlated in the patient data. Among these correlated variables, the one with the less significant *P*‐value in the univariate analysis and with more missing data (i.e., lymphatic invasion and adjuvant chemotherapy) were excluded from the multivariable analyses.

After these analyses, disease stage and microsatellite instability (MSI) status were the variables that remained significant in the baseline models for both OS and DFS. Although age was not significantly associated with OS in the univariate analysis, since it is a well‐established prognostic factor, it was included in the OS multivariable analyses.

Possible correlation between SNPs as well as the SNPs and categorical covariates in the multivariable models (i.e., stage and MSI status) was assessed by the Spearman's correlation test. The differences between the baseline characteristics of the discovery and replication cohorts were assessed using the Pearson's X^2^ test (categorical variables) or Mann–Whitney U test (age).

In the replication cohort, the univariate and multivariable Cox regression analyses for OS were applied to the top 16 SNPs using the same genetic model in the discovery cohort analysis. Within these 16 SNPs, in addition to the *MMP27*_rs11225388, *MMP27*_rs11225389, and *MMP8*_rs12365082, two other SNPs were in high linkage disequilibrium (LD) with each other (*VEGFC*_rs2877961 and *VEGFC*_rs7664413; Spearman's *r*_s_ = 0.985). *P*‐values reported in the replication cohort analyses are one‐sided.

Statistical analyses were conducted using IBM SPSS, (NY, USA) unless stated otherwise.

We computed the power of the 505 samples in the discovery cohort. Assuming a SNP in LD (D' =  1) with a risk allele frequency 0.3, we have at least 0.80 power to detect nominal significant association at *P* =  0.001 under a dominant model with moderate effect size of HR 1.55. For the validation cohort with 247 samples, to verify an association with the same assumptions and at *P* = 0.01 significance level, the statistical power is 0.81.

### Bioinformatics analyses

The rs numbers of *MMP27*_rs2846707, *MMP27*_rs11225388, *MMP27*_rs11225389, and *MMP8*_rs12365082, were entered in the RegulomeDB database 30 using the default conditions to recruit information about their potential regulatory functions. PolyPhen‐2 31 was used to estimate functional consequences for the amino acid substitution (Met30Val; rs2846707) in the MMP27 protein.

## Results

The results of the Cox univariate regression analysis for overall survival in the discovery cohort are summarized in Table S8.

After this analysis, 16 SNPs with the lowest *P*‐values (all *P* < 0.05) in the discovery cohort were genotyped and investigated in the replication cohort. As also shown in Table 2, out of 16 SNPs, only four SNPs had univariate analysis *P* < 0.05 in the validation cohort: *MMP27*_rs2846707, *MMP27*_rs11225388, *MMP27*_rs11225389, and *MMP8*_rs12365082. Therefore, these four SNPs were the only ones that had a significance value < 0.05 in both cohorts. According to our results, patients with the genotypes that contained the minor alleles (A allele in the cases of *MMP27*_rs2846707, *MMP27*_rs11225389, and *MMP8*_rs12365082, and G allele in the case of *MMP27*_rs11225388) had lower risk of death compared to the patients with the major allele homozygous genotypes. In both cohorts, the direction of effect was identical (i.e., reduced risk of death was associated with the minor allele homozygous and heterozygous genotypes). Figure S1 shows the Kaplan–Meier curves for these SNPs obtained in the discovery and replication cohorts.

**Table 2:** Cox univariate regression analysis results for the top 16 SNPs in the discovery and replication cohorts (overall survival)

Genetic Model	SNP	Discovery cohort					Replication cohort				
		* *P*‐value	HR	95% CI (lower)	95% CI (upper)	*n* (MAF)	** *P*‐value	HR	95% CI (lower)	95% CI (upper)	*n* (MAF)
Dom.	MMP27_rs11225388_A_G (GG + AG vs. AA)	**0.0005**	0.574	0.420	0.785	504 (27%)	**0.0162**	0.702	0.508	0.971	247 (25%)
Dom.	MMP27_rs11225389_C_A (AA + AC vs. CC)	**0.0005**	0.574	0.420	0.785	504 (27%)	**0.0127**	0.691	0.499	0.955	246 (25%)
Dom.	MMP8_rs12365082_T_A (AA + AT vs. TT)	**0.0006**	0.579	0.423	0.791	504 (27%)	**0.0125**	0.690	0.498	0.954	247 (24%)
Dom.	MMP25_rs1064948_T_A (AA + AT vs. TT)	**0.0013**	0.610	0.451	0.825	504 (37%)	0.1268	1.214	0.870	1.694	247 (39%)
Rec	MMP7_rs1996352_T_C (CC vs. TC + TT)	**0.0043**	2.438	1.322	4.495	504 (19%)	0.2636	0.726	0.269	1.960	247 (22%)
Rec	VEGFC_rs2877961_G_A (AA vs. AG + GG)	**0.0048**	2.333	1.295	4.202	504 (20%)	0.3853	1.119	0.524	2.391	247 (21%)
Rec	VEGFC_rs1485762_C_T (TT vs. TC + CC)	**0.0054**	1.814	1.193	2.758	504 (32%)	0.4625	1.025	0.619	1.697	247 (32%)
Dom.	MMP3_rs679620_A_G (GG + AG vs. AA)	**0.0061**	1.750	1.173	2.610	504 (49%)	0.4192	0.965	0.684	1.360	247 (47%)
Dom.	MMP27_rs2846707_G_A (AA + AG vs. GG)	**0.0064**	0.658	0.487	0.889	504 (36%)	**0.0411**	0.751	0.543	1.037	247 (38%)
Co‐Dom.	MMP16_rs7835845_C_T	**0.0094**				504 (25%)	0.3624				247 (24%)
	MMP16_rs7835845_C_T (CT vs. CC)	**0.0025**	1.609	1.183	2.188		0.2530	1.122	0.799	1.575	
	MMP16_rs7835845_C_T (TT vs. CC)	0.8161	1.096	0.506	2.377		0.2825	1.202	0.642	2.251	
Rec	VEGFC_rs7664413_C_T (TT vs. TC + CC)	**0.0148**	2.217	1.169	4.206	504 (20%)	0.3853	1.119	0.524	2.391	247 (20%)
Dom.	MMP16_rs16878625_T_C (CC + CT vs. TT)	**0.0150**	1.511	1.084	2.106	503 (11%)	0.1937	1.188	0.804	1.754	247 (9%)
Dom.	MMP16_rs2222294_C_T (TT + TC vs. CC)	**0.0161**	1.504	1.079	2.097	504 (12%)	0.1310	0.812	0.564	1.168	247 (15%)
Co‐Dom.	MMP16_rs2616487_A_G	**0.0171**				503 (33%)	0.4096				247 (43%)
	MMP16_rs2616487_A_G (AG vs. AA)	**0.0351**	0.697	0.498	0.975		0.4222	0.965	0.675	1.379	
	MMP16_rs2616487_A_G (GG vs. AA)	0.2730	1.269	0.829	1.941		0.2665	0.865	0.549	1.363	
Dom.	MMP16_rs4961076_T_C (CC + CT vs. TT)	**0.0176**	0.607	0.402	0.917	504 (11%)	0.3907	0.946	0.641	1.397	247 (12%)
Rec	MMP10_rs12272341_G_A (AA vs. AG + GG)	**0.0187**	2.663	1.177	6.023	504 (12%)	0.3586	1.294	0.321	5.224	247 (14%)

CI, confidence interval; Dom, dominant; HR, hazards ratio; MAF, minor allele frequency; n, number; MMP, Matrix Metalloproteinase; Rec, recessive. The first and the second letters after the *Gene*_SNP designations refer to the major and the minor alleles, respectively. *P*‐values less than 0.05 are bolded. *two‐sided *P*‐value; **one‐sided *P*‐value.

The genomic region containing the *MMP27*_rs2846707, *MMP27*_rs11225388, *MMP27*_rs11225389, and *MMP8*_rs12365082 polymorphisms is depicted in Figure S2. Three SNPs were in almost complete linkage disequilibrium in both the discovery and replication patient cohorts (*MMP27*_rs11225388, *MMP27*_rs11225389, and *MMP8*_rs12365082; the minimum Spearman's r_s_ between any two polymorphisms in either cohort was 0.99), while *MMP27*_rs2846707 had a somewhat different profile (*r*_s_ = 0.8 and 0.68–0.69 in the discovery and replication cohorts, respectively). They are also common polymorphisms with minor allele frequencies of 24–28% (Table 2). As of May 9, 2016 there were no literature reports about these SNPs. Only one of these SNPs substitute the amino acids encoded by these genes; *MMP27*_rs2846707 (NP_071405.2:p.Met30Val); this polymorphisms was predicted as a benign substitution by PolyPhen‐2 31. According to the dbSNP 32 and RegulomeDB 30 databases, *MMP8*_rs12365082 is located in the 3′‐UTR of *MMP8* and with no potential regulatory function noted; and *MMP27*_rs11225388 and *MMP27*_rs11225389 are located in an intron and 5′‐UTR of *MMP27*, respectively, both with minimal regulatory or protein‐binding potential. There was no RegulomeDB data available for *MMP27*_rs2846707.

Univariate analyses were followed by the multivariable analysis of the top 16 SNPs (Table 3). When adjusted for stage, MSI status, and age at diagnosis, there were 10 SNPs, including *MMP27*_rs11225388, *MMP27*_rs11225389, and *MMP8*_rs12365082 polymorphisms (but not *MMP27*_rs2846707), that showed an independent association with overall survival in the discovery cohort. However, in the analysis of replication cohort, no significance was detected for any of the 16 SNPs examined (Table 3). Also, none of the SNPs in Table 2 were correlated with stage or MSI in either the discovery or the replication cohorts.

**Table 3:** Cox multivariable regression analysis results for the top 16 SNPs in the discovery and replication cohorts (overall survival)

Genetic Model	Gene_SNP	Discovery cohort					Replication cohort				
		* *P*‐value	HR	95% CI (lower)	95% CI (upper)	*n*	** *P*‐value	HR	95% CI (lower)	95% CI (upper)	*n*
Dom.	*MMP27*_rs11225388_A_G (GG + AG vs. AA)	**0.0013**	0.589	0.426	0.814	483	0.2730	0.903	0.647	1.259	240
Dom.	*MMP27*_rs11225389_C_A (AA + AC vs. CC)	**0.0013**	0.589	0.426	0.814	483	0.2230	0.878	0.629	1.226	239
Dom.	*MMP8*_rs12365082_T_A (AA + AT vs. TT)	**0.0016**	0.594	0.430	0.821	483	0.2320	0.883	0.632	1.232	240
Dom.	*MMP25*_rs1064948_T_A (AA + AT vs. TT)	**0.0404**	0.722	0.529	0.986	483	0.3350	1.077	0.766	1.513	240
Rec.	*MMP7*_rs1996352_T_C (CC vs. TC + TT)	0.1723	1.568	0.822	2.993	483	0.3325	0.801	0.294	2.186	240
Rec.	*VEGFC*_rs2877961_G_A (AA vs. AG + GG)	**0.0029**	2.589	1.383	4.847	483	0.3409	0.851	0.394	1.840	240
Rec.	*VEGFC_*rs1485762_C_T (TT vs. TC + CC)	**0.0281**	1.624	1.054	2.503	483	0.4282	0.954	0.570	1.596	240
Dom.	*MMP3*_rs679620_A_G (GG + AG vs. AA)	**0.0300**	1.573	1.045	2.368	483	0.3858	0.949	0.669	1.347	240
Dom.	*MMP27*_rs2846707_G_A (AA + AG vs. GG)	0.0512	0.731	0.534	1.002	483	0.4644	1.015	0.728	1.416	240
Co‐dom.	*MMP16*_rs7835845_C_T	0.0630				483	0.4747				240
	*MMP16*_rs7835845_C_T (CT vs. CC)	0.5032	1.309	0.595	2.877		0.4461	0.976	0.688	1.385	
	*MMP16*_rs7835845_C_T (TT vs. CC)	0.1292	1.832	0.838	4.004		0.3982	1.089	0.569	2.085	
Rec.	*VEGFC*_rs7664413_C_T (TT vs. TC + CC)	**0.0194**	2.263	1.141	4.489	483	0.3409	0.851	0.394	1.840	240
Dom.	*MMP16*_rs16878625_T_C (CC + CT vs. TT)	0.3698	1.173	0.828	1.661	482	0.3164	1.102	0.740	1.643	240
Dom.	*MMP16*_rs2222294_C_T (TT + TC vs. CC)	0.3257	1.194	0.839	1.700	483	0.1758	0.837	0.575	1.217	240
Co‐dom.	*MMP16*_rs2616487_A_G	0.1015				482	0.2960				240
	*MMP16*_rs2616487_A_G (AG vs. AA)	**0.0325**	0.687	0.487	0.969		0.3525	1.075	0.739	1.564	
	*MMP16*_rs2616487_A_G (GG vs. AA)	0.5187	0.861	0.545	1.358		0.2486	0.848	0.527	1.365	
Dom.	*MMP16*_rs4961076_T_C (CC + CT vs. TT)	0.2532	0.780	0.509	1.195	483	0.4971	1.002	0.667	1.505	240
Rec.	*MMP10*_rs12272341_G_A (AA vs. AG + GG)	**0.0135**	2.852	1.242	6.546	483	0.0857	2.684	0.652	11.048	240

CI, confidence interval; Dom, dominant; HR, hazards ratio; n, number; MMP, Matrix Metalloproteinase; Rec, recessive. The first and the second letters after the *Gene*_SNP designations refer to the major and the minor alleles, respectively. *P*‐values less than 0.05 are bolded. Each SNP was adjusted for age at diagnosis, disease stage, and MSI status. *two‐sided *P*‐value; **one‐sided *P*‐value.

As a secondary analysis, we also performed Cox univariate regression analysis for disease‐free survival in the discovery cohort (Table S8; the results for the top 16 SNPs are also tabulated in Table S9). Interestingly, the lowest *P*‐values in this analysis were obtained for the *MMP27*_rs11225388 and *MMP27*_rs11225389 polymorphisms. Similar to the OS analysis, the minor allele‐containing genotypes were associated with the reduced risk of recurrence, metastasis, or death in the discovery cohort (*P* = 0.002, HR: 0.637, 95% CI: 0.478–0.848 for both polymorphisms). When adjusted for stage and MSI, similar results were obtained (*P* = 0.002, HR: 0.630, 95% CI: 0.469–0.846; Table S9).

Finally, the comparison of the baseline characteristics used for adjustment in the multivariable analyses showed that the median age of diagnosis in the replication cohort was significantly higher than the discovery cohort (*P* < 0.001), whereas the proportions of stage (*P* = 0.054) and MSI (*P* = 0.492) were comparable between the two cohorts. We hypothesized that the age differences between the two cohorts could be the reason for the nonreplication of the discovery cohort results in the multivariable modeling of the replication cohort. Thus, we repeated the analysis for the patients ≤75 years of age in the replication cohort (similar to the discovery cohort; *n* = 164). Again, significance levels could not be reached for the *MMP27*_rs2846707, *MMP27*_rs11225388, *MMP27*_rs11225389, and *MMP8*_rs12365082 polymorphisms (*data not shown*).

## Discussion

It is well recognized that the induction of angiogenesis (generation of new blood vessels) and lymph‐angiogenesis (generation of new lymphatic vessels) around the solid tumors have critical roles in their growth and invasive character, and thus the disease progression. Unfortunately, these processes are also linked to metastasis as they provide the vascular and lymphatic routes for tumor cells to move to and form metastases at distant sites 2, 33, 34. Metastasis is a multi‐step and complex process requiring alterations in the tumor microenvironment, particularly degradation of the extracellular matrix that serves as a physiological barrier for the tumor cell invasion. Not surprisingly, progressed and metastatic cancers are more difficult to manage compared to localized/early stage cancers, almost always associated with poor patient prognosis, and responsible for the majority of the cancer‐related deaths. These highlight the importance of examining the angiogenesis, lymph‐angiogenesis, and metastasis pathway genes in cancer survival studies.

These three biological processes are regulated by a number of genes, among which the Vascular Endothelial Growth Factor (VEGF) and Matrix Metalloproteinase (MMP) families are the most studied ones. VEGF ligands and receptors interact with each other to initiate signaling cascades and binding of different VEGF ligands and receptors leads to different intracellular response 1. For example, binding of VEGFA to FLT1/VEGFR1 or KDR/VEGFR2 receptors promotes angiogenesis. On the other hand, lymph‐angiogenesis requires binding of VEGFC or VEGFD molecules to another VEGF receptor, FLT4/VEGFR3. Because of their established roles in cancer progression, there have been efforts to target VEGF molecules in treatment of cancer patients. Bevacizumab, a monoclonal anti‐VEGFA antibody used in treatment of colorectal and other cancers is a well‐known example 35.

Considering these established roles of VEGFs and MMPs in cancer progression and metastasis, in this study, we aimed to test the associations of 381 polymorphisms from the main VEGF and MMP genes and the risk of death (overall survival analysis) in two cohorts of colorectal cancer patients. Our main result is that none of the associations of SNPs in the discovery cohort was replicated in the replication cohort (Table 2 and Table 3). These results suggest that either the associations in the discovery cohort were false‐positive findings or the two cohorts are not comparable/significantly different from each other. Considering this latter possibility, we repeated the multivariable analysis for the most promising four SNPs (*MMP27*_rs2846707, *MMP27*_rs11225388, *MMP27*_rs11225389, and *MMP8*_rs12365082) in the replication cohort patients, who were ≤75 years of age at the time of diagnosis (similar to the discovery cohort patients). But this did not change the results, although we cannot rule out the insufficient study power in this subcohort, either. Overall, our conclusion is that, our study does not support a prognostic role for the examined VEGFA and MMP gene polymorphisms in colorectal cancer.

Many genes included in our study have been previously studied in colorectal cancer in relation to a variety of survival outcomes 19, 20, 36, 37, 38, 39. Similar to our study, some of the polymorphisms in our set were also examined as candidate markers of overall survival in colorectal cancer by multivariable analyses and using genotypes from germline (i.e., nontumor) DNAs. For example, the two well‐known *VEGFA* SNPs, namely ‐634G/C (rs2010963) and +936C/T (rs3025039) were associated with overall survival in a study by Dassoulas et al. 9. Yet, neither the presented study nor other studies 38, 40 have identified prognostic associations of these polymorphisms with overall survival. The inconsistent results may be due to the differences in study design/ethnicity, sample size/study power, or the baseline and treatment characteristics of the patient cohorts in different studies. For the rest of the polymorphisms, a comparison of the polymorphisms curated in the dbCPCO database 41 indicated that none of them were previously examined in relation to overall or disease‐free survivals in colorectal cancer patients with similar treatment characteristics to ours (i.e., not treated with bevacizumab). Considering the importance of the *VEGFA* gene and to check whether under different genetic models we would identify associations of its SNPs, we repeated the univariate Cox regression analyses under all genetic models for the 11 *VEGFA* gene SNPs included in our study. This analysis showed that one of the SNPs (rs3024994; NM_001025366.2:c.658 + 1378C>T) had a *P*‐value of 0.972, 0.024, 0.036, and 0.071 in recessive, dominant, additive, and codominant genetic models, respectively. The smallest *P*‐value obtained for this SNP was under the dominant genetic model (*P* = 0.024; the same model that this SNP was investigated in our study), yet, this p‐value was not small enough to be within the top 16 SNPs selected for replication purposes (the p‐values of which ranged from 0.0005 to 0.0187; Table 2). According to PUBMED as of May 2016, this SNP has not been investigated in relation to colorectal cancer survival before and can be a good candidate to investigate in other cohorts. Other 10 SNPs had univariate Cox analysis *P* > 0.05 under all four genetic models (data not shown).

Like any other study, this study too has some strengths and limitations. One notable strength is that we have had an independent replication cohort to test the reliability of the statistical findings in the discovery cohort. This is also the first study that has comprehensively examined a large number of polymorphisms from the VEGF ligand, VEGF receptor, and matrix metalloproteinase genes in relation to survival outcomes in colorectal cancer. Additionally, almost all of the polymorphisms in this study were examined in relation to overall survival in colorectal cancer for the first time. As per limitations, our approach was limited to the common variants (minor allele frequencies ≥5%) with available genotypes in the study populations; thus, we may have missed rare genetic variations that could have strong effects on the risk of outcome or many common variations that are not included in the genotyping platform. Large‐scale variant identification in the patient cohort, such as by means of next generation sequencing, may help identify the complete set of variants in these genes for future survival studies. Also, we did not investigate all of the genes known to function in the angiogenesis, lymph‐angiogenesis, and metastasis pathways. Therefore, prognostic studies may be expanded to other genes acting in these pathways, such as the tissue inhibitors of metalloproteinases (TIMPs) 42, to comprehensively examine their potential roles in patient survival. Finally, the discovery and validation cohort of patients had differences in their clinical characteristics; thus, the nonreplication of the associations in the validation cohort, which were detected in the discovery cohort, may be attributed to these differences. Also, both the study cohorts were treated with mostly 5‐FU‐based chemotherapy; thus, our findings cannot be generalized to patient cohorts treated with anti‐VEGF drugs such as bevacizumab.

In conclusion, as one of the most common cancers 43), identification of additional prognostic biomarkers may help with better prognostication and clinical management of colorectal cancer patients worldwide. The presented study investigated a large number of common polymorphisms from the members of the VEGF and MMP gene families that can modify the risk of disease progression and patient mortality. While our study did not find an evidence of association of the examined polymorphisms and overall survival in colorectal cancer, we cannot rule out the possible replication of the prognostic associations of the polymorphisms shown in Table 3 (particularly, the *MMP27_*rs11225388, *MMP27_*rs11225389, *MMP27*_rs2846707, and *MMP8*_rs1236508 polymorphisms) and Table S9 in larger and more powered study cohorts.

## Conflict of Interest

None declared.

## Supporting information


**Figure S1.** Kaplan–Meier survival plots for the four polymorphisms with p < 0.05 in both the discovery (A) and replication (B) cohorts.

**Figure S2.** The MMP gene cluster on chromosome 11q22.

**Table S1.** Genes and the number of SNPs from each gene investigated during this study.

**Table S2.** Kaplan‐Meier curves for VEGF and VEGFR SNPs (overall survival analysis).

**Table S3.** Kaplan‐Meier curves for MMP1‐MMP16 SNPs (overall survival analysis).

**Table S4.** Kaplan‐Meier curves for MMP17‐MMP28 SNPs (overall survival analysis).

**Table S5.** Kaplan‐Meier curves for VEGF SNPs (disease‐free survival analysis).

**Table S6.** Kaplan‐Meier curves for MMP1‐MMP16 SNPs (disease‐free survival analysis).

**Table S7.** Kaplan‐Meier curves for MMP17‐MMP28 SNPs (disease‐free survival analysis).

**Table S8.** Results of the univariate Cox regression analysis for the selected SNPs (overall survival and disease survival analyses).

**Table S9.** (A) Cox univariate analysis (top 16 SNPs), (B) Cox multivariable regression analysis (top 16 SNPs).
