# Supplementary material for: No associations of a set of SNPs in the Vascular Endothelial Growth Factor (VEGF) and Matrix Metalloproteinase (MMP) genes with survival of colorectal cancer patients
Source: Cancer Med. 2016 Jun 23;5(9):2221–31. doi: 10.1002/cam4.796 (PMC5055182; doi:10.1002/cam4.796)
Supplement: Supplementary file 1 — Figure S1. Kaplan–Meier survival plots for the four polymorphisms with p < 0.05 in both the discovery (A) and replication (B) cohorts. [file CAM4-5-2221-s001.pdf]

Figure 1: Kaplan-Meier survival plots for the four polymorphisms with $p < 0.05$ in both the discovery (A) and replication (B) cohorts

A) Discovery cohort (overall survival)

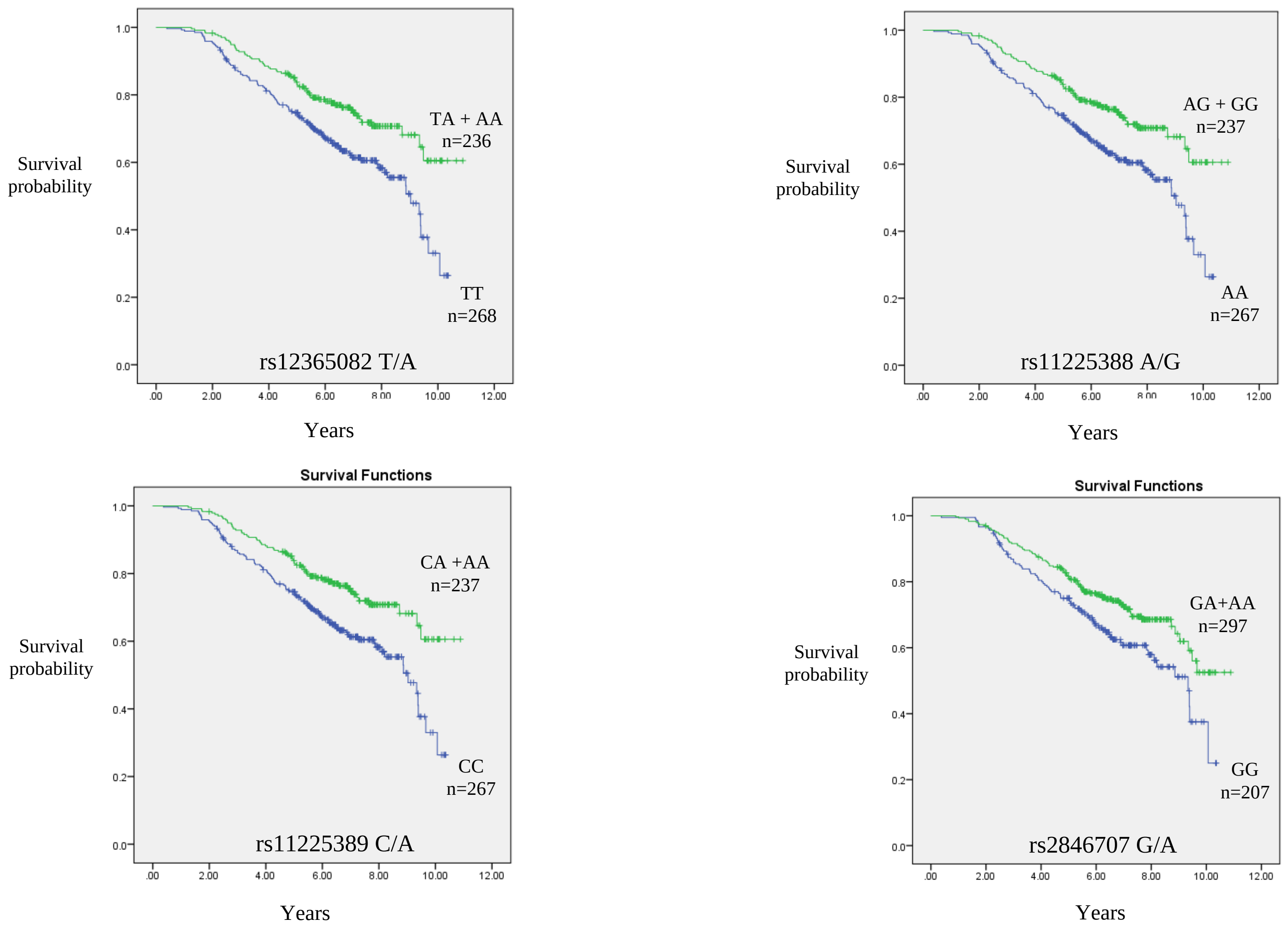

B) Replication cohort (overall survival)

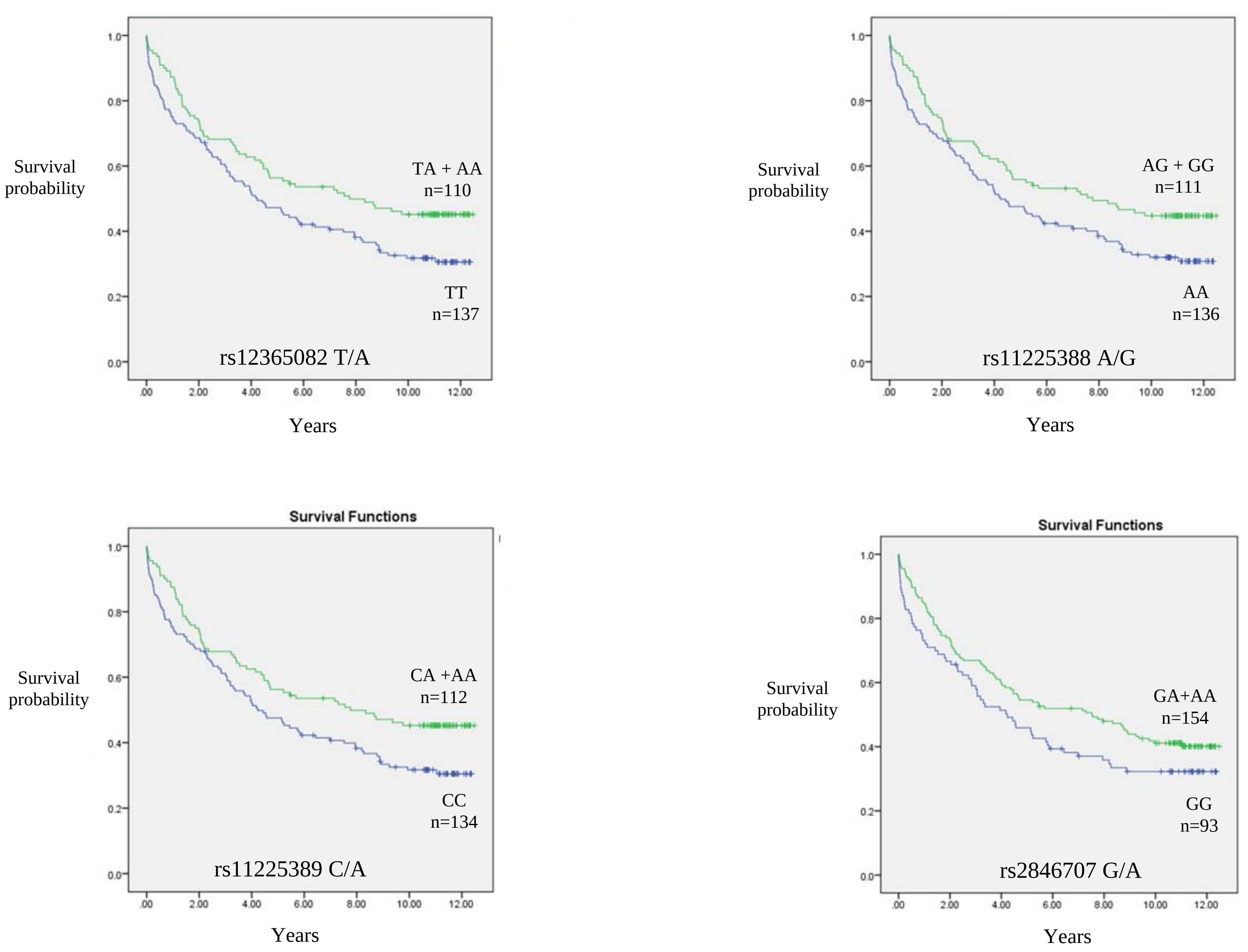

Kaplan Meier curves for the four highly linked polymorphisms that had the lowest univariate analysis p-values in both the discovery and the replication cohorts.
