# Supplementary material for: No associations of a set of SNPs in the Vascular Endothelial Growth Factor (VEGF) and Matrix Metalloproteinase (MMP) genes with survival of colorectal cancer patients
Source: Cancer Med. 2016 Jun 23;5(9):2221–31. doi: 10.1002/cam4.796 (PMC5055182; doi:10.1002/cam4.796)
Supplement: Supplementary file 2 — Figure S2. The MMP gene cluster on chromosome 11q22. [file CAM4-5-2221-s002.pdf]

Supplementary Figure 2. The MMP gene cluster on chromosome 11q22

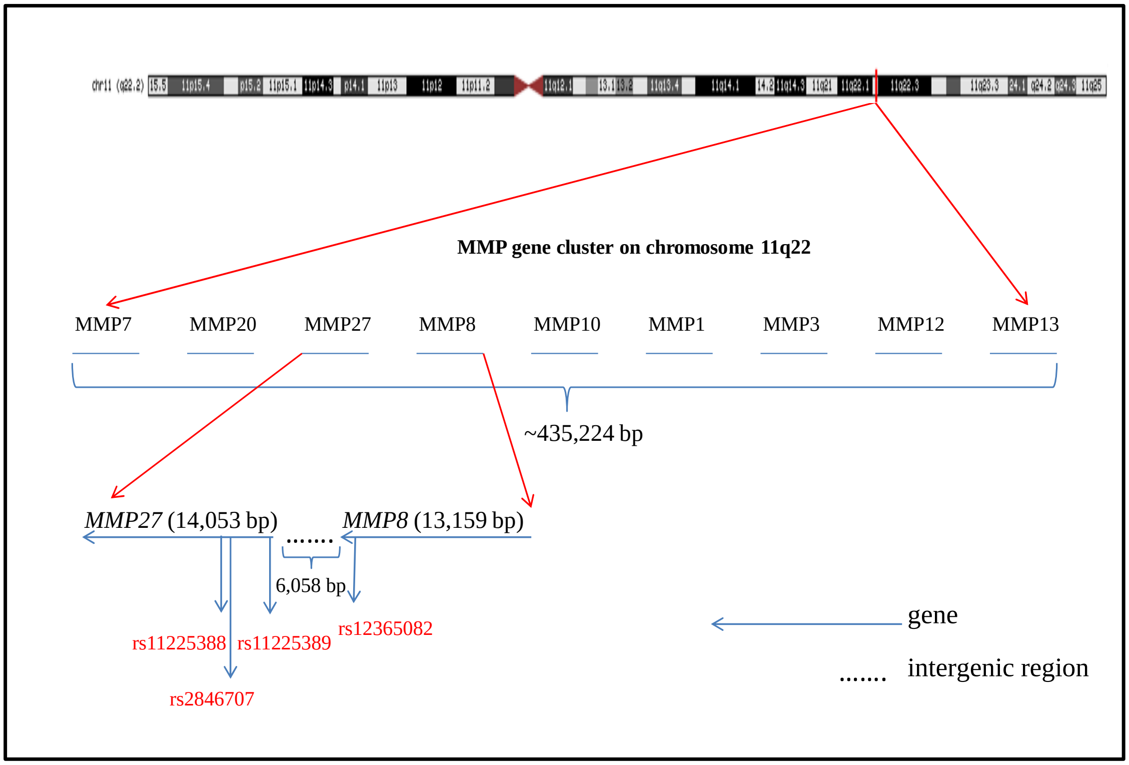

The MMP gene cluster on chromosome 11q22 encompasses ~ 435 kilobases long region and contains nine MMP genes. *MMP27* and *MMP8* genes are 14,053 and 13,159 base pairs long, respectively. The distance between the *MMP27*_rs11225388 and *MMP27*_rs2846707, *MMP27*_rs11225389, and *MMP8*_rs12365082 polymorphisms are 110, 729 and 7,093 base pairs, respectively. Chromosomal bar is obtained from the UCSC genome browser website (25). Figure not drawn to scale.
