# Supplementary material for: No associations of a set of SNPs in the Vascular Endothelial Growth Factor (VEGF) and Matrix Metalloproteinase (MMP) genes with survival of colorectal cancer patients
Source: Cancer Med. 2016 Jun 23;5(9):2221–31. doi: 10.1002/cam4.796 (PMC5055182; doi:10.1002/cam4.796)
Supplement: Supplementary file 3 — Table S1. Genes and the number of SNPs from each gene investigated during this study. [file CAM4-5-2221-s003.pdf]

Supplementary Information

Supplementary Table 1. Genes and the number of SNPs from each gene investigated during this study.

**Gene**	**Number of SNPs**
VEGFA	11
VEGFB	2
VEGFC	19
VEGFD	7
PGF	2
VEGFR1	49
VEGFR2	19
VEGFR3	20
MMP1	10
MMP2	22
MMP3	4
MMP7	5
MMP8	9
MMP9	6
MMP10	11
MMP11	3

MMP12	3
MMP13	4
MMP14	9
MMP15	4
MMP16	70
MMP17	13
MMP19	3
MMP20	21
MMP21	3
MMP23B	0
MMP24	25
MMP25	7
MMP26	1
MMP27	17
MMP28	2
**Total = 31**	**Total = 381**
